# Supplementary material for: X-ray Radiotherapy Impacts Cardiac Dysfunction by Modulating the Sympathetic Nervous System and Calcium Transients
Source: Int J Mol Sci. 2024 Aug 31;25(17):9483. doi: 10.3390/ijms25179483 (PMC11394929; doi:10.3390/ijms25179483)
Supplement: Supplementary file 1 [file ijms-25-09483-s001.zip › ijms-3169338-supplementary.pdf]

Supplementary data Figure S1 : Phosphorylation of H2AX at Ser 139 (γ-H2AX) immunolabeling

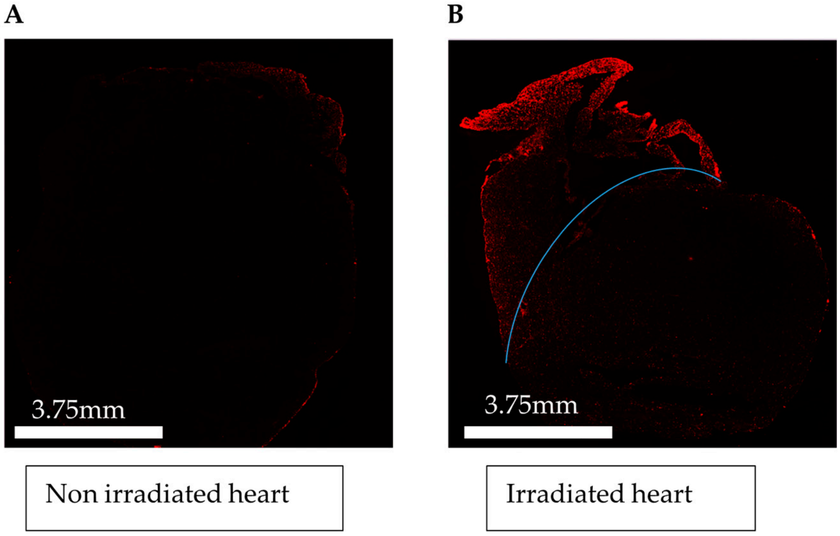

Figure S1 : Phosphorylation of H2AX at Ser 139 (γ-H2AX) immunolabeling 24h post Irradiation.A. P-H2AX is present mainly in the atria and a small upper part of the ventricle in irradiated hearts, in contrast to NIR (B).

SUPPLEMENTARY Data Table S1 : VEVO parameters

		NIR	0.25Gy	0.5Gy	2Gy
	N	6	6	6	6
LVEF (%)	Mean ± SEM	77.80 ± 1.09	70.41 ± 2.61	67.20 ± 2.65	65.00 ± 2.81
	p-value	-	0.102	* 0.0142	** 0.0033
LVFS (%)	Mean ± SEM	45.75 ± 1.18	39.69 ± 2.19	37.08 ± 2.06	35.36 ± 1.99
	p-value	-	0.087	* 0.0112	** 0.0026
Stroke volume (µL)	Mean ± SEM	36.96 ± 3.35	46.58 ± 4.64	42.99 ± 2.05	45.65 ± 2.69
	p-value	-	0.13	0.45	0.19
Cardiac output (mL/min)	Mean ± SEM	21.75 ± 3.03	22.07 ± 2.63	21.78 ± 1.48	23.95 ± 1.40
	p-value	-	0.99	> 0.99	0.83
LVs volume (µL)	Mean ± SEM	13.21 ± 0.95	20.18 ± 2.71	20.81 ± 2.54	26.49 ± 3.02
	p-value	-	0.14	0.1	** 0.0028
LVd volume (µL)	Mean ± SEM	46.49 ± 4.73	52.63 ± 5.68	52.27 ± 4.54	59.07 ± 3.83
	p-value	-	0.69	0.72	0.18
LWs depth (mm)	Mean ± SEM	1.49 ± 0.10	1.45 ± 0.04	1.32 ± 0.05	1.27 ± 0.06
	p-value	-	0.96	0.18	0.079
LWd depth (mm)	Mean ± SEM	1.13 ± 0.06	1.02 ± 0.01	0.91 ± 0.04	0.91 ± 0.03
	p-value	-	* 0.046	** 0.002	** 0.0025
LVIDs depth (mm)	Mean ± SEM	2.02 ± 0.06	2.38 ± 0.13	2.41 ± 0.12	2.66 ± 0.12
	Stats	-	0.089	0.06	** 0.0017
LVIDd depth (mm)	Mean ± SEM	3.35 ± 0.14	3.35 ± 0.14	3.52 ± 0.16	3.72 ± 0.10
	p-value	-	0.7	0.71	0.17
IVSd/LWd	Mean ± SEM	0.93 ± 0.10	0.92 ± 0.06	0.91 ± 0.09	1.01 ± 0.08
	p-value	-	0.99	0.99	0.85
LV mass/BW	Mean ± SEM	4.64 ± 0.38	3.84 ± 0.24	3.34 ± 0.15	3.99 ± 0.24
	p-value	-	0.12	** 0.0065	0.23
AoV flow VTI (mm)	Mean ± SEM	26.29 ± 2.21	34.21 ± 4.53	28.88 ± 2.96	26.45 ± 1.32
	p-value	-	0.42	> 0.99	> 0.99
MV flow VTI (mm)	Mean ± SEM	15.51 ± 1.18	14.29 ± 2.30	15.31 ± 1.89	14.85 ± 1.34
	p-value	-	0.92	0.99	0.99
MV E/A	Mean ± SEM	1.86 ± 0.16	1.74 ± 0.25	1.61 ± 0.08	1.50 ± 0.07
	p-value	-	0.90	0.50	0.24

Table S1: All VEVO parameters analyzed. Data were presented as mean ± SEM (n = 6 per group). * $p < 0.05$, ** $p < 0.01$ vs. NIR mice by one-way ANOVA test followed by Dunnett's multiple comparison post hoc test

Supplementary data Figure S2 : Explanation of the filament tracer (IMARIS)

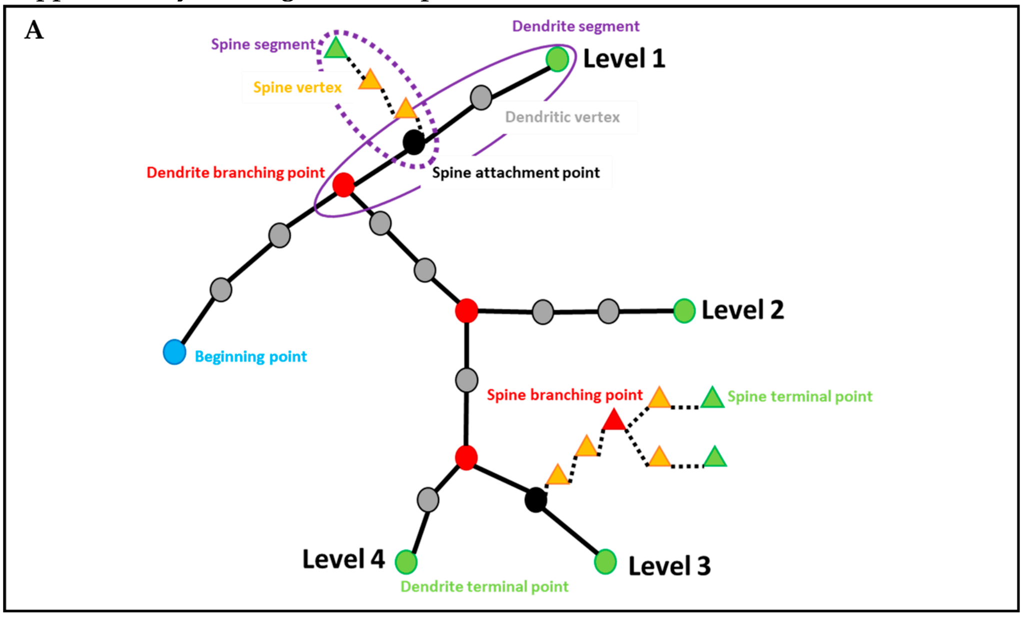

Figure S2 : Schematic representation of the algorithms for automated tracing of sympathetic nervous system filaments immunostaining by Tyrosine Hydroxylase (TH) in imaris software. Full lines and circles represent dendritic edges and dendritic vertices respectively. Dotted lines and triangles represent spine edges and spines vertices respectively.
